# Editorial: Inflammatory disorders of the oral mucosa: current challenges and future perspectives

**DOI:** 10.3389/froh.2024.1497187

**Published:** 2024-09-27

**Authors:** Victor Desmond Mandel, Guya Diletta Marconi, Jacopo Pizzicannella, Alessia Paganelli

**Affiliations:** ^1^ Porphyria and Rare Diseases Unit, San Gallicano Dermatological Institute-IRCCS, Rome, Italy; ^2^ Department of Innovative Technologies in Medicine and Dentistry, University “G. D'Annunzio” Chieti-Pescara, Chieti, Italy; ^3^ Dipartimento di Ingegneria e Geologia, University “G. D'Annunzio” Chieti-Pescara, Chieti, Italy; ^4^ Clinical Dermatology Unit, IDI- IRCCS Istituto Dermopatico Dell'Immacolata, Rome, Italy

**Keywords:** inflammatory diseases, oral cavity, mucosal immunity, lichen, autoimmune blistering dermatoses

**Editorial on the Research Topic**
Inflammatory disorders of the oral mucosa: from stem cell biology to clinical management

The oral cavity has a peculiar anatomical and immunological milieu and is commonly affected by inflammatory conditions (1). These include a diverse group of disorders, stemming from a range of causes, including infections, autoimmune or purely inflammatory forms. Clinically, a wide range of signs and symptoms can accompany inflammatory disorders of the oral mucosa, including erythema, erosions, discomfort, pain, and impaired oral function. Understanding the precise underlying mechanisms of these conditions is essential for accurate diagnosis and treatment.

The field of research surrounding inflammatory disorders of the oral mucosa is rapidly expanding due to advances in immunology, molecular biology, and diagnostic technologies. Researchers are increasingly focusing on understanding the complex interactions between the immune system, microbiome, and environmental factors that contribute to the pathogenesis of oral inflammation. Key areas of interest include the role of dysbiosis (microbial imbalance) in triggering inflammation, the impact of genetic predispositions on disease susceptibility, and the identification of specific biomarkers that could aid in early diagnosis and targeted therapies.

Novel therapeutic approaches are also being explored, such as biologics that modulate immune responses and regenerative techniques to repair damaged mucosal tissues (2). Additionally, the growing understanding of links between oral inflammation and systemic conditions like cardiovascular disease, diabetes, and autoimmune disorders is broadening the scope of research (3, 4). This interdisciplinary approach is paving the way for more personalized and effective treatments, enhancing both oral and overall health outcomes (5).

A central position in the scenario of inflammatory oral disorders is certainly occupied by autoimmune blistering diseases. In fact, oral involvement is particularly common in this subset of dermatological morbid conditions. Mucous membrane pemphigoid (MMP) represents one of the most severe progressive forms potentially affecting the oral mucosa Jakubowska et al. Early diagnosis and prompt treatment are crucial to prevent possible complications, including scarring and functional impairment. The oral cavity is frequently involved in patients with MMP (approximately 90% of cases), either in combination or not with other mucous membranes. Ocular involvement is rarer but potentially leading to visual loss in up to 75% of cases. The gold standard for diagnosis is direct immunofluorescence (DIF). Recent data indicate that oral DIF is positive in 100% of patients with oral involvement, but also in the vast majority of cases with ocular involvement, both isolated and associated with oral MMP. These results highlight that oral biopsy is usually sufficient for the diagnosis, even in patients with exclusively ocular MMP.

Despite not as common as in pemphigus vulgaris, oral blisters are also a possible feature of bullous pemphigoid (BP). However, HSV infection is common in BP oral lesions and, therefore, the differential diagnosis of oral blisters in patients affected by BP is particularly challenging. The absence of skin lesions, presence of pain, the concomitant use of high-dose glucocorticoid should alert physicians to HSV infection in oral lesions and treat them with systemic antiviral treatment timely. However, oral lesions in course of BP may also be part of associated syndromic clinical pictures, as described in a recent report of a case of secondary Sjogren syndrome in a patient affected by BP treated with dupilumab Chen et al.

Another important group of oral inflammatory diseases is represented by oral lichen planus (OLP). OLP often presents as white, lacy patches, painful sores, or red, swollen areas. The exact cause of OLP is not fully understood, but it is thought to involve an immune-mediated response to mucosal tissues. OLP can cause discomfort, burning sensations, and difficulty in eating or speaking, significantly affecting a patient's quality of life. Despite low, a risk of malignant transformation has been described for OLP. Topical and/or intralesional corticosteroids are currently considered the first-line treatment of OLP. However, alternative strategies have been introduced for the management of OLP in the last decades, including immunosuppressants, systemic corticosteroids, biologics and low-level laser therapy. An updated systematic review and meta-analysis of 17 randomized clinical trials has recently been conducted with the aim of assessing whether clobetasol propionate could be considered the gold standard for patients affected by OLP; the results of the study supported the long-term application of CLO as an effective regimen in OLP patients Zheng et al.

Lichen sclerosus (LS) is a chronic inflammatory condition predominantly affecting the anogenital region, with extragenital manifestations being relatively rare (6). Despite uncommon, oral lichen sclerosus (OLS) is a possible presentation, often under-recognized due to its asymptomatic nature and rarity in clinical practice Paganelli et al. Clinically, OLS often presents as asymptomatic whitish macules or plaques predominantly affecting the labial, gingival and palatal mucosa. Histopathological examination typically reveals subepithelial hyalinization, loss of elastic fibers, and a band-like inflammatory infiltrate. Treatment with topical corticosteroids and/or intralesional triamcinolone acetonide injections was effective in managing the lesions. Given its rarity, increasing awareness among healthcare providers is crucial for early detection.

**Figure 1:**
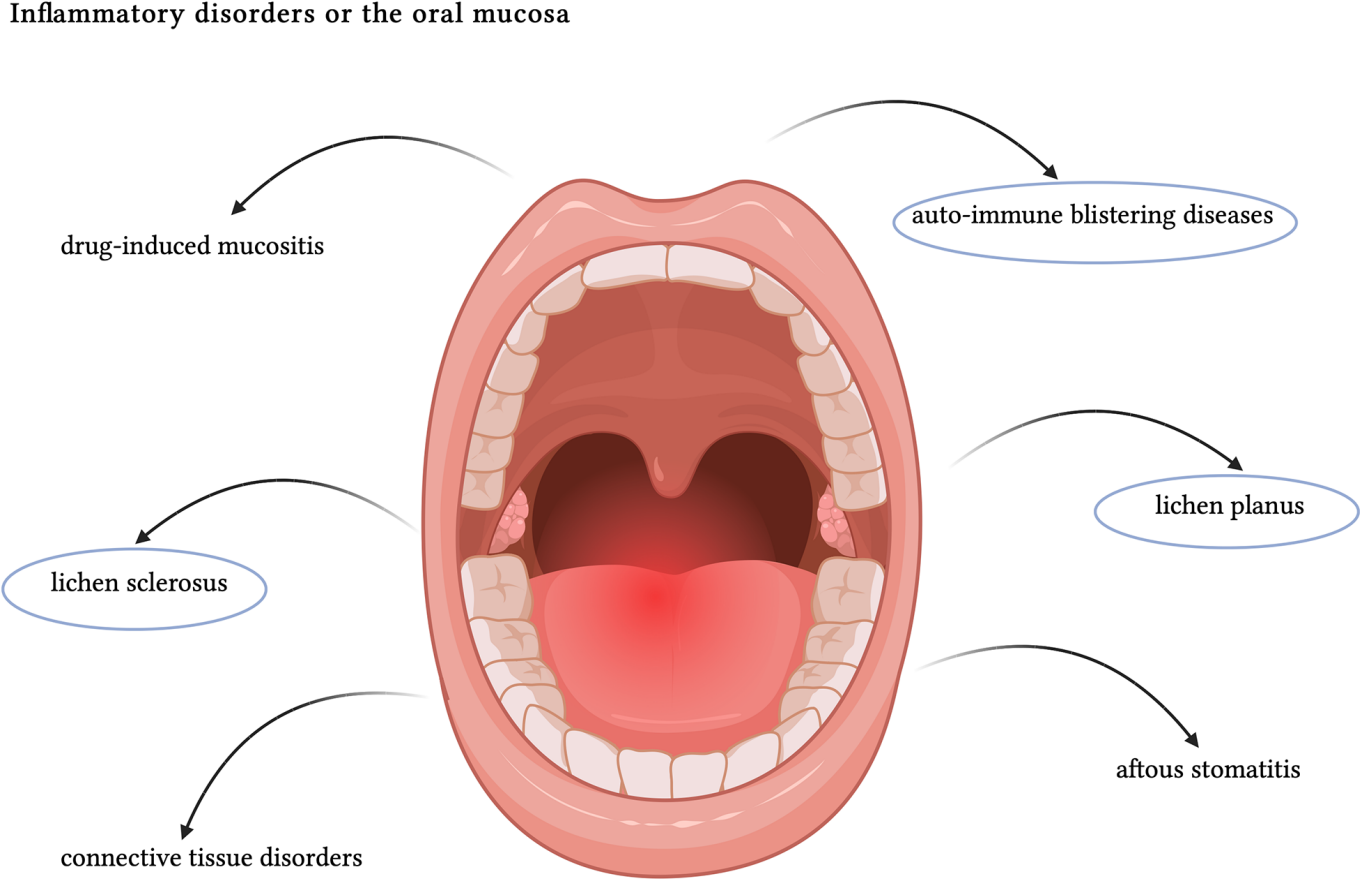
Schematic representation of the main causes of oral inflammation. The main themes of the present article have been circled in blue. Created with BioRender.com.

Despite not exhaustive of all the potential disorders affecting the oral mucosa, the present research topic provides an overview of oral involvement in the setting of autoimmune blistering diseases, oral lichen planus, and oral lichen sclerosus (Figure 1). The integration of clinical and histopathological findings is crucial for accurate diagnosis and effective management of these conditions. Advances in scientific research are paving the way for better understanding of their pathogenesis and optimization of therapeutic strategies.
